# Updated Rice Kinase Database RKD 2.0: enabling transcriptome and functional analysis of rice kinase genes

**DOI:** 10.1186/s12284-016-0106-5

**Published:** 2016-08-19

**Authors:** Anil Kumar Nalini Chandran, Yo-Han Yoo, Peijian Cao, Rita Sharma, Manoj Sharma, Christopher Dardick, Pamela C Ronald, Ki-Hong Jung

**Affiliations:** 1 Graduate School of Biotechnology & Crop Biotech Institute, Kyung Hee University, Yongin, 446-701 Republic of Korea; 2 China Tobacco Gene Research Center, Zhengzhou Tobacco Research Institute, Zhengzhou, 450001 China; 3 School of Computational & Integrative Sciences, Jawaharlal Nehru University, New Delhi, 110067 India; 4 School of Biotechnology, Jawaharlal Nehru University, New Delhi, 110067 India; 5 Appalachian Fruit Research Station, United States Department of Agriculture – Agricultural Research Service, 2217 Wiltshire Road, Kearneysville, WV 25442 USA; 6 Department of Plant Pathology and the Genome Center, University of California, Davis, CA 95616 USA; 7 The Joint Bioenergy Institute, Emeryville, CA 95616 USA

**Keywords:** Rice kinase database, Phylogenomics, Functional redundancy, Mutant analysis, Meta-analysis, Pearson correlation coefficient, Transcriptome

## Abstract

**Background:**

Protein kinases catalyze the transfer of a phosphate moiety from a phosphate donor to the substrate molecule, thus playing critical roles in cell signaling and metabolism. Although plant genomes contain more than 1000 genes that encode kinases, knowledge is limited about the function of each of these kinases. A major obstacle that hinders progress towards kinase characterization is functional redundancy. To address this challenge, we previously developed the rice kinase database (RKD) that integrated omics-scale data within a phylogenetics context.

**Results:**

An updated version of rice kinase database (RKD) that contains metadata derived from NCBI GEO expression datasets has been developed. RKD 2.0 facilitates in-depth transcriptomic analyses of kinase-encoding genes in diverse rice tissues and in response to biotic and abiotic stresses and hormone treatments. We identified 261 kinases specifically expressed in particular tissues, 130 that are significantly up- regulated in response to biotic stress, 296 in response to abiotic stress, and 260 in response to hormones. Based on this update and Pearson correlation coefficient (PCC) analysis, we estimated that 19 out of 26 genes characterized through loss-of-function studies confer dominant functions. These were selected because they either had paralogous members with PCC values of <0.5 or had no paralog.

**Conclusion:**

Compared with the previous version of RKD, RKD 2.0 enables more effective estimations of functional redundancy or dominance because it uses comprehensive expression profiles rather than individual profiles. The integrated analysis of RKD with PCC establishes a single platform for researchers to select rice kinases for functional analyses.

**Electronic supplementary material:**

The online version of this article (doi:10.1186/s12284-016-0106-5) contains supplementary material, which is available to authorized users.

## Background

Protein kinases are involved in diverse cellular and biological processes. Elucidation of their roles is limited; functions for only 61 kinases in rice (*Oryza sativa*) have been ascribed (Yamamoto et al. 2012). This subset represents approximately 4.1 % of all kinase genes in that genome. Since sequencing of the entire rice genome was completed in 2005 (IRGSP 2005), diverse omics datasets have been generated that include genome-wide expression analysis using microarrays as well as RNA-seq, re-sequencing of 3000 rice accessions, protein–protein interactomes, gene regulatory networks, and metabolic pathways. Because these databases use very different platforms for formatting, it has been a challenge to integrate publicly available datasets to facilitate functional genomics studies (Chandran and Jung 2014; Alexandrov et al. 2015).

Functional redundancy is a significant obstacle to the identification of gene functions. Completed whole-genome sequences enable researchers to estimate the extent of functional redundancy due to gene duplications. From 20 to 60 % of the rice genome is tandemly or segmentally duplicated (Itoh et al. 2007; Lin et al. 2008). In addition, 21,403 proteins in rice have been assigned to 3856 paralogous protein family groups, thus demonstrating that more than 50 % of all rice genes annotated to non-transposable element (non-TE) gene models have functional redundancy because of their paralogous relationships (Lin et al. 2008). However, not all paralogous genes have such redundancy. Knock-out mutant analysis has identified light response-defective phenotypes in T-DNA insertional mutant lines for nine gene family members that are predominantly expressed under high illumination (Jung et al. 2008). In addition, 79 of 127 ubiquitously expressed genes which have been functionally characterized through loss-of-function studies belong to gene families (Jung et al. 2015). In both cases, functional dominance of the characterized genes within a particular family has been readily estimated through phylogenomics analyses that integrate metadata from diverse expression datasets within a phylogenetics context (Jung et al. 2015).

To date, six phylogenomics databases have been developed for rice. These include kinase, glycosyltransferase, glycoside hydrolase, transcription factor, transporter, and cytochrome p450 databases (Jung et al. 2015). Rice kinase database (RKD) was the first of these to be established, and it has provided a basic framework for all others (Dardick et al. 2007; Jung et al. 2010). Application of RKD in facilitating functional genomic studies was previously demonstrated. For example, phylogenomics approach played a major role in the identification of a set of MAPK, MAPKK, and MAPKKK genes that are part of same signaling cascades, and in predicting the regulatory model of a light-inducible kinase gene (Jung et al. 2010).

One unique feature of RKD compared with other databases is an option to integrate protein–protein interaction network data based on yeast two-hybrid or tandem affinity purification tagging analyses (Dardick et al. 2007). A large set of this integrated transcriptomic data, retrieved from the National Center for Biotechnology Information Gene Expression Omnibus (NCBI GEO; www.ncbi.nlm.nih.gov/geo/. accessed on November 21, 2015), is a main provider of diverse biological information about interesting kinase genes for further functional studies (Cao et al. 2008; Jung et al. 2010). The current format of those databases for integrating transcriptome data has proven cumbersome because users must mine data from a list of datasets within NCBI GEO. However, Genevestigator, RiceXPro, and Rice Oligonucleotide Array Database (ROAD; www.ricearray.org/, accessed on December 2, 2015) have recently integrated these meta-expression data through classification and reconstruction to enhance accessibility (Zimmermann et al. 2008; Cao et al. 2012; Sato et al. 2013).

Here, we describe RKD 2.0, an updated RKD that integrates metadata from genes expressed in particular tissues and in response to abiotic/biotic stresses and hormone treatments. Using these data, a genome-wide meta-analysis of expression patterns for all predicted rice kinases have been performed and identified kinase-encoding genes with distinct expression patterns. Also, steps involved in the construction and application of RKD 2.0 has been discussed.

## Results and discussion 

### Updated features of RKD 2.0

The earlier version, RKD, presented phylogenomics data for 1508 kinases comprising 65 subfamilies, and a group of kinase genes that could not be assigned to any of those subfamilies (Dardick et al. 2007; Jung et al. 2010). Because that version utilized all gene models (splicing variants), the integrated transcriptome information showed redundant expression data for multiple models produced from a particular locus. However, for the current version, we selected one representative model per locus based on the information available from rice genome annotation project (RGAP) database (Ouyang et al. 2007) that could provide a simplified and comprehensive visualization of the integrated data. Likewise, instead of taking individual datasets from NCBI GEO (Barrett et al. 2011), Affymetrix-based anatomical metadata (ROAD; http://www.ricearray.org/expression/experiment_search.php, accessed on December 2, 2015), used in ROAD (Cao et al. 2012) have been integrated within the phylogenic tree context. Furthermore, meta-analysis with diverse datasets of genes that are expressed in response to biotic and abiotic stresses as well as hormones treatment was performed. Unlike the average normalized intensity data used for an anatomical meta-expression database, log_2_ fold-change data for treatment versus control have been introduced. Differential expression in response to five pathogens (*Magnaporthe grisea*, MG; *Magnaporthe oryzae*, MO; rice stripe virus, RSV; *Xanthomonas oryzae* pv. *oryzae*, Xoo; and the brown plant hopper, BPH), in addition to five abiotic stresses (drought, salt, cold, heat, and submergence) and six hormones (abscisic acid, ABA; brassinolide, BL; gibberellic acid, GA; indole-3-acetic acid, IAA; trans-zeatin, tZ; and jasmonic acid, JA) were also analyzed. The results are presented in Additional files 1, 2, 3, 4 and 5: Tables S1-S5. The expression data used for meta-analysis from anatomical tissues and in stress responses were based on Affymetrix array data downloaded from the NCBI GEO (Cao et al. 2012; Zimmermann et al. 2008). For hormone responses, those expression data were based on Agilent 44 K arrays (GSE39429) (NCBI GEO; http://www.ncbi.nlm.nih.gov/geo/query/acc.cgi?acc=GSE39429, accessed on November 21, 2015) (Sato et al. 2013). As an alternative platform for meta-analysis of microarray data, RNASeq data of anatomical and abiotic stress treated samples were integrated to the database (Secco et al. 2013; Wang et al. 2015). All of these updated features are available in the new rice kinase database (RKD 2.0; http://ricephylogenomics-khu.org/kinase/index.php, accessed on February 20, 2016).

### Meta-analysis of expression data available for kinase genes with known functions

Using information from OGRO, the Overview of Functionally Characterized Genes in Rice online database (OGRO; http://qtaro.abr.affrc.go.jp/ogro/table. Accessed 20 October 2015) (Yamamoto et al. 2012), 61 RGAP loci related to kinases that have been functionally characterized were retrieved. Among them, function of a subset of kinase genes were related to more than one trait. Thus, 61 kinases are associated with 124 traits. Although these genes were broadly classified as conferring morphological traits (35 genes), physiological traits (27), stress responses resistance (59), or other biological functions (3) (Table 1), 63 of them are associated with multiple functions. Among those for morphological traits, 10 genes each target for culm leaves and dwarfism. Another three genes each are related to panicle/flower formation and seeds, four to roots and five to seedling development. The major physiological effects of rice mutants lead to sterility (6 genes), indicating that the genes in this category are important for fertilization and embryo development. The physiological-trait classification also includes four genes related to source activity, six for flowering, two for eating quality, and one for seed production. In that group, mutations of three genes are attributed to germination dormancy. In addition to the panicle-dependent effect, physiological effects can be observed in the roots (two genes) and culm leaves (one gene). Mutation of one gene causes lethality. The stress-response classification is subdivided into genes that function in biotic stresses, abiotic stresses, and resistance to mechanical stresses such as lodging. With regard to biotic stresses, 15 genes have roles in blast resistance, eight including* Xa21 *gene involved in bacterial blight resistance (Song et al. 1995) , function of a gene associated with insect resistance and one in resistance to other diseases. For abiotic stresses, eight genes are associated with drought, 15 with salinity, six with chilling, and another five with other types of stresses. Analysis of previously characterized genes indicates that kinases plays diverse role in physiology and growth of rice plants.

**Table 1:** Functional information for kinase genes with known functions

Type of meta-expression	Character_major	LOC_id	Gene name	Gene symbol	Method	character_minor	DOI reference
IAA	Morphological trait	LOC_Os01g52050.1	dwarf 61	d61	Mutant	Culm leaf	10.1105/tpc.12.9.1591
Anther/Pollen	Morphological trait	LOC_Os02g58610.1	N-acetylglucosaminyltransferase I	GnTI	Mutant	Culm leaf	10.1111/tpj.12087
Root, BPH	Morphological trait	LOC_Os06g50920.1	increased leaf angle1	ila1	Mutant	Culm leaf	10.1105/tpc.111.093419
	Morphological trait	LOC_Os02g05980.1	leucine-rich repeat receptor-like kinase1	LRK1	Overexpression	Culm leaf	10.1111/j.1467-7652.2009.00428.x
	Morphological trait	LOC_Os02g05980.1	Leucine-rich repeat receptor-like kinase 1	LRK1	Overexpression	Culm leaf	10.1007/s10529-012-1054-9
	Morphological trait	LOC_Os02g40860.1	Low Temperature Growth 1	LTG1	Others	Culm leaf	10.1111/tpj.12487
	Morphological trait	LOC_Os08g07760.1	OsBAK1	OsBAK1	Knockdown	Culm leaf	10.1111/j.1467-7652.2009.00444.x
	Morphological trait	LOC_Os05g40770.1	Leucine-rich-repeat receptor-like kinases	OsRPK1	Knockdown Overexpression	Culm leaf	10.1016/j.bbagen.2014.01.003
	Morphological trait	LOC_Os02g32610.2	CONSTITUTIVE TRIPLE-RESPONSE2	OsCTR2	Mutant	Culm leaf	10.1093/jxb/ert272
	Morphological trait	LOC_Os04g48760.1	xiao	xiao	Mutant	Culm leaf	10.1111/j.1365-313X.2011.04877.x
IAA	Morphological trait	LOC_Os01g52050.1	dwarf 61	d61	Mutant	Dwarf	10.1105/tpc.12.9.1591
Anther/Pollen	Morphological trait	LOC_Os02g58610.1	N-acetylglucosaminyltransferase I	GnTI	Mutant	Dwarf	10.1111/tpj.12087
	Morphological trait	LOC_Os02g05980.1	leucine-rich repeat receptor-like kinase1	LRK1	Overexpression	Dwarf	10.1111/j.1467-7652.2009.00428.x
	Morphological trait	LOC_Os02g05980.1	Leucine-rich repeat receptor-like kinase 1	LRK1	Overexpression	Dwarf	10.1007/s10529-012-1054-9
	Morphological trait	LOC_Os02g40860.1	Low Temperature Growth 1	LTG1	Others	Dwarf	10.1111/tpj.12487
Leaf/Flag leaf/Shoot, ABA	Morphological trait	LOC_Os02g36570.1	activity of bc1 complex 1–2	OsABC1-2	Mutant	Dwarf	10.1016/j.gene.2012.02.017
	Morphological trait	LOC_Os08g07760.1	OsBAK1	OsBAK1	Knockdown	Dwarf	10.1111/j.1467-7652.2009.00444.x
	Morphological trait	LOC_Os02g03410.1	Ca2 + −dependent protein kinase 4	OsCPK4	Knockdown	Dwarf	10.1104/pp.113.230268
	Morphological trait	LOC_Os05g40770.1	Leucine-rich-repeat receptor-like kinases	OsRPK1	Knockdown Overexpression	Dwarf	10.1016/j.bbagen.2014.01.003
	Morphological trait	LOC_Os04g48760.1	xiao	xiao	Mutant	Dwarf	10.1111/j.1365-313X.2011.04877.x
	Morphological trait	LOC_Os06g50340.1	FLORAL ORGAN NUMBER1	fon1	Mutant	Panicle flower	10.1242/dev.01441
	Morphological trait	LOC_Os02g05980.1	leucine-rich repeat receptor-like kinase1	LRK1	Overexpression	Panicle flower	10.1111/j.1467-7652.2009.00428.x
SAM/Panicle, IAA	Morphological trait	LOC_Os12g42020.1	pinoid	OsPID	Overexpression	Panicle flower	10.1093/pcp/pcm024
	Morphological trait	LOC_Os02g14120.1	defective in outer cell layer specification 1	Docs1	Mutant	Root	10.1111/j.1365-313X.2011.04824.x
	Morphological trait	LOC_Os02g40860.1	Casein Kinase I	OsCKI1	Knockdown	Root	10.1046/j.1365-313X.2003.01866.x
SAM/Panicle, IAA	Morphological trait	LOC_Os12g42020.1	pinoid	OsPID	Overexpression	Root	10.1093/pcp/pcm024
	Morphological trait	LOC_Os05g40770.1	Leucine-rich-repeat receptor-like kinases	OsRPK1	Knockdown Overexpression	Root	10.1016/j.bbagen.2014.01.003
Leaf/Flag leaf/Shoot, ABA	Morphological trait	LOC_Os02g36570.1	activity of bc1 complex 1–2	OsABC1-2	Mutant	Seed	10.1016/j.gene.2012.02.017
Ubiquitous, JA	Morphological trait	LOC_Os03g03660.1	calcium-dependent protein kinase 1	OsCDPK1	Knockdown Overexpression	Seed	10.1007/s11103-012-0006-z
	Morphological trait	LOC_Os04g48760.1	xiao	xiao	Mutant	Seed	10.1111/j.1365-313X.2011.04877.x
Ubiquitous, JA	Morphological trait	LOC_Os03g03660.1	calcium-dependent protein kinase 1	OsCDPK1	Knockdown Overexpression	Shoot seedling	10.1007/s11103-012-0006-z
tZ	Morphological trait	LOC_Os03g20380.1	Calcineurin B-like protein-interacting protein kinases 31	oscipk31	Mutant	Shoot seedling	10.1007/s10059-010-0084-1
JA	Morphological trait	LOC_Os08g42750.1	Calcium-dependent protein kinase21	OsCPK21	Overexpression	Shoot seedling	10.1007/s11103-010-9717-1
	Morphological trait	LOC_Os02g32610.2	CONSTITUTIVE TRIPLE-RESPONSE2	OsCTR2	Mutant	Shoot seedling	10.1093/jxb/ert272
	Morphological trait	LOC_Os05g45420.1	Snf1-related protein kinases 1a	snrk1a	Mutant	Shoot seedling	10.1105/tpc.105.037887
SAM/Panicle, IAA	Physiological trait	LOC_Os07g32480.1	Bub1-Related Kinase 1	BRK1	Mutant	Sterility	10.1105/tpc.112.105874
RSV, IAA	Physiological trait	LOC_Os01g68870.1	MULTIPLE SPOROCYTE 1	msp1	Mutant	Sterility	10.1105/tpc.012401
Root	Physiological trait	LOC_Os07g05620.1	CBL (Calcineurin B-Like) Interacting Protein Kinase 23	OsCIPK23	Knockdown Overexpression	Sterility	10.1016/S1673-8527(08)60073-9
JA	Physiological trait	LOC_Os09g38850.1	DEFECT IN EARLY EMBRYO SAC1	OsDEES1	Knockdown	Sterility	10.1104/pp.112.203943
	Physiological trait	LOC_Os07g08000.1	NIMA-related kinase 3	OsNek3	Overexpression	Sterility	10.1093/pcp/pcp026
	Physiological trait	LOC_Os04g48760.1	xiao	xiao	Mutant	Sterility	10.1111/j.1365-313X.2011.04877.x
Anther/Pollen	Physiological trait	LOC_Os02g58610.1	N-acetylglucosaminyltransferase I	GnTI	Mutant	Source activity	10.1111/tpj.12087
Leaf/Flag leaf/Shoot, ABA	Physiological trait	LOC_Os02g36570.1	activity of bc1 complex 1–2	OsABC1-2	Mutant	Source activity	10.1016/j.gene.2012.02.017
Leaf/Flag leaf/Shoot	Physiological trait	LOC_Os05g40180.1	A serine/threo- nine protein kinase 8	OsSTN8	Mutant	Source activity	10.1111/tpj.12331
	Physiological trait	LOC_Os11g01140.1	Phototropin1a	phot1a	Mutant	Source activity	10.1007/s11103-008-9442-1
	Physiological trait	LOC_Os03g57940.1	earlier flowering1	el1	Mutant	Flowering	10.1038/emboj.2010.75
	Physiological trait	LOC_Os03g57940.1	Heading date 16	Hd16	Natural variation	Flowering	10.1111/tpj.12268
	Physiological trait	LOC_Os03g55389.1	heading date 6	Hd6	Natural variation	Flowering	10.1073/pnas.111136798
	Physiological trait	LOC_Os02g40860.1	Low Temperature Growth 1	LTG1	Others	Flowering	10.1111/tpj.12487
	Physiological trait	LOC_Os03g56270.1	NUTRITION RESPONSE AND ROOT GROWTH b	NRRb	Knockdown	Flowering	10.1093/mp/sss157
	Physiological trait	LOC_Os02g32610.2	CONSTITUTIVE TRIPLE-RESPONSE2	OsCTR2	Mutant	Flowering	10.1093/jxb/ert272
	Physiological trait	LOC_Os10g39420.1	seed-specific protein kinase	SPK	Knockdown	Eating quality	10.1093/pcp/pch122
	Physiological trait	LOC_Os10g39420.1	seed-specific protein kinase	SPK	Knockdown	Eating quality	10.1105/tpc.010454
tZ	Physiological trait	LOC_Os03g20380.1	Calcineurin B-like protein-interacting protein kinases 31	oscipk31	Mutant	Germination dormancy	10.1007/s10059-010-0084-1
	Physiological trait	LOC_Os04g12540.1	lectin receptor kinase	OslecRK	Knockdown	Germination dormancy	10.1111/tpj.12328
	Physiological trait	LOC_Os05g45420.1	Snf1-related protein kinases 1a	snrk1a	Mutant	Germination dormancy	10.1105/tpc.105.037887
	Physiological trait	LOC_Os05g40770.1	Leucine-rich-repeat receptor-like kinases	OsRPK1	Knockdown Overexpression	Seed	10.1016/j.bbagen.2014.01.003
	Physiological trait	LOC_Os05g41090.1	Ca2 + calmodulin-de-pendent protein kinase	OsCCaMK	Mutant	Root	10.1128/AEM.03646-13.
	Physiological trait	LOC_Os02g03410.1	Ca2 + −dependent protein kinase 4	OsCPK4	Overexpression	Root	10.1104/pp.113.230268
Root	Physiological trait	LOC_Os05g40770.1	Leucine-rich-repeat receptor-like kinases	OsRPK1	Knockdown Overexpression	Culm leaf	10.1016/j.bbagen.2014.01.003
	Physiological trait	LOC_Os02g40860.1	hybrid breakdown 2	hbd2	Natural variation	Lethality	10.1007/s00438-010-0514-y
	Physiological trait	LOC_Os02g05480.1	TEY-type rice mitogen-activated protein kinase 3	OsMPK3	Others	Others	10.1007/s00299-014-1620-9
Cold	Resistance or Tolerance	LOC_Os09g36320.1	BROAD-SPECTRUM RESISTANCE 1	BSR1	Overexpression	Blast resistance	10.1111/j.1467-7652.2010.00568.x
Heat, JA	Resistance or Tolerance	LOC_Os07g22710.1	calcium-dependent protein kinase (CDPK) 18	CPK18	Knockdown	Blast resistance	10.1105/tpc.114.126441
	Resistance or Tolerance	LOC_Os10g40100.1	receptor-like cytoplasmic kinase gene	NRRB	Knockdown	Blast resistance	10.1007/s11033-014-3069-x
BPH	Resistance or Tolerance	LOC_Os03g06410.1	accelerated cell death and resistance 1	OsACDR1	Knockdown Overexpression	Blast resistance	10.1007/s10059-009-0161-5
	Resistance or Tolerance	LOC_Os03g12730.1	blast resistance-related 1	OsBRR1	Knockdown Overexpression	Blast resistance	10.1007/s00425-009-0951-1
JA	Resistance or Tolerance	LOC_Os08g42580.1	chitin elicitor receptor kinase1	OsCERK1	Knockdown	Blast resistance	10.1111/j.1365-313X.2010.04324.x
Cold	Resistance or Tolerance	LOC_Os03g57450.1	Calcium-dependent protein kinase 10	OsCPK10	Overexpression	Blast resistance	10.1016/j.plaphy.2013.10.004
	Resistance or Tolerance	LOC_Os04g47300.1	Calcium-dependent protein kinase 12	OsCPK12	Mutant	Blast resistance	10.1111/j.1365-313X.2011.04766.x
	Resistance or Tolerance	LOC_Os04g12540.1	lectin receptor kinase	OslecRK	Knockdown	Blast resistance	10.1111/tpj.12328
	Resistance or Tolerance	LOC_Os03g17700.1	Mitogen-activated protein kinase5	OsMAPK5	Knockdown Overexpression	Blast resistance	10.1105/tpc.008714
	Resistance or Tolerance	LOC_Os02g05480.1	TEY-type rice mitogen-activated protein kinase 3	OsMPK3	Others	Blast resistance	10.1007/s00299-014-1620-9
Cold	Resistance or Tolerance	LOC_Os04g41160.1	oxidative signal inducible 1	OsOxi1	Overexpression	Blast resistance	10.1093/pcp/pcq132
	Resistance or Tolerance	LOC_Os01g65230.1	3-phosphoinositide-dependent protein kinase 1	OsPdk1	Mutant	Blast resistance	10.1093/pcp/pcq167
Cold	Resistance or Tolerance	LOC_Os04g38480.1	somatic embryogenesis receptor-like kinase 1	OsSERK1	Overexpression	Blast resistance	10.1007/s00425-005-1534-4
	Resistance or Tolerance	LOC_Os06g29810.1	Pi-d2	Pi-d2	Natural variation	Blast resistance	10.1111/j.1365-313X.2006.02739.x
Cold	Resistance or Tolerance	LOC_Os09g36320.1	BROAD-SPECTRUM RESISTANCE 1	BSR1	Overexpression	Bacterial blight resistance	10.1111/j.1467-7652.2010.00568.x
BPH	Resistance or Tolerance	LOC_Os03g06410.1	enhanced disease resistance 1	OsEDR1	Mutant	Bacterial blight resistance	10.1111/j.1365-3040.2010.02219.x
	Resistance or Tolerance	LOC_Os04g12540.1	lectin receptor kinase	OslecRK	Knockdown	Bacterial blight resistance	10.1111/tpj.12328
	Resistance or Tolerance	LOC_Os03g17700.1	Mitogen-activated protein kinase5	OsMAPK5	Knockdown Overexpression	Bacterial blight resistance	10.1105/tpc.008714
	Resistance or Tolerance	LOC_Os10g38950.1	Mitogen-Activated Protein Kinase Phosphatase6	OsMPK6	Mutant	Bacterial blight resistance	10.1007/s00425-007-0541-z
Cold, JA	Resistance or Tolerance	LOC_Os04g41160.1	oxidative signal inducible 1	OsOxi1	Overexpression	Bacterial blight resistance	10.1093/pcp/pcq132
	Resistance or Tolerance	LOC_Os01g65230.1	3-phosphoinositide-dependent protein kinase 1	OsPdk1	Mutant	Bacterial blight resistance	10.1093/pcp/pcq167
	Resistance or Tolerance	LOC_Os11g35500.1	Xa21	Xa21	Natural variation	Bacterial blight resistance	10.1126/science.270.5243.1804
	Resistance or Tolerance	LOC_Os03g17700.1	Mitogen-activated protein kinase5	OsMAPK5	Knockdown Overexpression	Other disease resistance	10.1105/tpc.008714
	Resistance or Tolerance	LOC_Os04g12540.1	lectin receptor kinase	OslecRK	Knockdown	Insect resistance	10.1111/tpj.12328
Cold	Resistance or Tolerance	LOC_Os02g50970.1	drought-hypersensitive mutant1	dsm1	Mutant	Drought tolerance	10.1104/pp.109.149856
JA	Resistance or Tolerance	LOC_Os03g03660.1	calcium-dependent protein kinase 1	OsCDPK1	Knockdown Overexpression	Drought tolerance	10.1007/s11103-012-0006-z
Cold	Resistance or Tolerance	LOC_Os04g49510.1	calcium-dependent protein kinase 7	OsCDPK7	Overexpression	Drought tolerance	10.1046/j.1365-313x.2000.00787.x
Drought/Salt, M. grisea, ABA	Resistance or Tolerance	LOC_Os01g55450.1	calcineurin B-like protein-interacting protein kinase12	OsCIPK12	Overexpression	Drought tolerance	10.1104/pp.107.101295
Cold, M. oryzae	Resistance or Tolerance	LOC_Os02g03410.1	Ca2 + −dependent protein kinase 4	OsCPK4	Knockdown	Drought tolerance	10.1104/pp.113.230268
	Resistance or Tolerance	LOC_Os01g10840.1	glycogen synthase kinase3-like gene 1	OsGSK1	Mutant	Drought tolerance	10.1007/s11103-007-9213-4
	Resistance or Tolerance	LOC_Os03g17700.1	Mitogen-activated protein kinase5	OsMAPK5	Knockdown Overexpression	Drought tolerance	10.1105/tpc.008714
	Resistance or Tolerance	LOC_Os06g03970.1	stress-induced protein kinase gene 1	sik1	Mutant	Drought tolerance	10.1111/j.1365-313X.2010.04146.x
Cold	Resistance or Tolerance	LOC_Os04g49510.1	calcium-dependent protein kinase 7	OsCDPK7	Overexpression	Salinity tolerance	10.1046/j.1365-313x.2000.00787.x
BPH	Resistance or Tolerance	LOC_Os11g02240.1	calcineurin B-like protein-interacting protein kinase15	OsCIPK15	Overexpression	Salinity tolerance	10.1104/pp.107.101295
	Resistance or Tolerance	LOC_Os07g05620.1	CBL (Calcineurin B-Like) Interacting Protein Kinase 23	OsCIPK23	Knockdown Overexpression	Salinity tolerance	10.1016/S1673-8527(08)60073-9
tZ	Resistance or Tolerance	LOC_Os03g20380.1	Calcineurin B-like protein-interacting protein kinases 31	oscipk31	Mutant	Salinity tolerance	10.1007/s10059-010-0084-1
	Resistance or Tolerance	LOC_Os04g47300.1	Calcium-dependent protein kinase 12	OsCPK12	Mutant	Salinity tolerance	10.1111/j.1365-313X.2011.04766.x
JA	Resistance or Tolerance	LOC_Os08g42750.1	Calcium-dependent protein kinase21	OsCPK21	Overexpression	Salinity tolerance	10.1007/s11103-010-9717-1
Cold, M. oryzae	Resistance or Tolerance	LOC_Os02g03410.1	Ca2 + −dependent protein kinase 4	OsCPK4	Knockdown	Salinity tolerance	10.1104/pp.113.230268
	Resistance or Tolerance	LOC_Os01g10840.1	glycogen synthase kinase3-like gene 1	OsGSK1	Mutant	Salinity tolerance	10.1007/s11103-007-9213-4
	Resistance or Tolerance	LOC_Os02g05480.1	Mitogen-activated protein kinase33	OsMAPK33	Overexpression	Salinity tolerance	10.1007/s12038-011-9002-8
	Resistance or Tolerance	LOC_Os03g17700.1	Mitogen-activated protein kinase5	OsMAPK5	Knockdown Overexpression	Salinity tolerance	10.1105/tpc.008714
	Resistance or Tolerance	LOC_Os06g05520.1	mitogen-activated protein kinase kinase	OsMKK1 (MAPKK)	Knockdown	Salinity tolerance	10.1016/j.plantsci.2014.08.007
ABA	Resistance or Tolerance	LOC_Os01g64970.1	SNF1-type serine-threonine protein kinase4	SAPK4	Overexpression	Salinity tolerance	10.1186/1471-2229-8-49
	Resistance or Tolerance	LOC_Os06g03970.1	stress-induced protein kinase gene 1	sik1	Mutant	Salinity tolerance	10.1111/j.1365-313X.2010.04146.x
JA	Resistance or Tolerance	LOC_Os02g42780.1	Salt Intolerance 1	SIT1	Knockdown Overexpression	Salinity tolerance	10.1105/tpc.114.125187
Cold	Resistance or Tolerance	LOC_Os04g44900.1	Salt Intolerance 2	SIT2	Knockdown	Salinity tolerance	10.1105/tpc.114.125187
JA	Resistance or Tolerance	LOC_Os03g03660.1	calcium-dependent protein kinase 13	CDPK13	Overexpression	Cold tolerance	10.1007/s00438-007-0220-6
JA	Resistance or Tolerance	LOC_Os03g03660.1	calcium-dependent protein kinase 13	OsCDPK13	Overexpression	Cold tolerance	10.1007/s11103-004-1178-y
tZ	Resistance or Tolerance	LOC_Os03g20380.1	calcineurin B-like protein-interacting protein kinase03	OsCIPK03	Overexpression	Cold tolerance	10.1104/pp.107.101295
	Resistance or Tolerance	LOC_Os01g10840.1	glycogen synthase kinase3-like gene 1	OsGSK1	Mutant	Cold tolerance	10.1007/s11103-007-9213-4
Cold	Resistance or Tolerance	LOC_Os03g17700.1	Mitogen-activated protein kinase5	OsMAPK5	Knockdown Overexpression	Cold tolerance	10.1105/tpc.008714
Cold, M. oryzae, IAA	Resistance or Tolerance	LOC_Os01g32660.1	Oryza sativa MAPK kinase 6	OsMKK6	Overexpression	Cold tolerance	10.1016/j.jplph.2011.11.012
Cold	Resistance or Tolerance	LOC_Os02g50970.1	drought-hypersensitive mutant1	dsm1	Mutant	Other stress resistance	10.1104/pp.109.149856
ABA	Resistance or Tolerance	LOC_Os02g36570.1	activity of bc1 complex 1–2	OsABC1-2	Mutant	Other stress resistance	10.1016/j.gene.2012.02.017
Heat	Resistance or Tolerance	LOC_Os05g41090.1	Doesn’t make infection 3	OsDMI3	Mutant	Other stress resistance	10.1093/mp/sss068
	Resistance or Tolerance	LOC_Os01g10840.1	glycogen synthase kinase3-like gene 1	OsGSK1	Mutant	Other stress resistance	10.1007/s11103-007-9213-4
Cold	Resistance or Tolerance	LOC_Os04g41160.1	oxidative signal inducible 1	OsOxi1	Overexpression	Other stress resistance	10.1093/pcp/pcq132
tZ	Others	LOC_Os08g34380.1	commissural vein excessive1	coe1	Mutant	Others	10.1111/j.1365-313X.2010.04250.x
Cold	Others	LOC_Os08g40170.1	plant-specific cyclin-dependent kinase2;1	Orysa;CDKB2;1	Knockdown	Others	10.1111/j.1365-313X.2011.04847.x
Cold, JA	Others	LOC_Os06g06090.1	Mitogen-activated protein kinase6	OsMAPK6	Knockdown	Others	10.1104/pp.104.057414

### Functional assignment of rice kinase genes using expression data

**Fig. 1:**
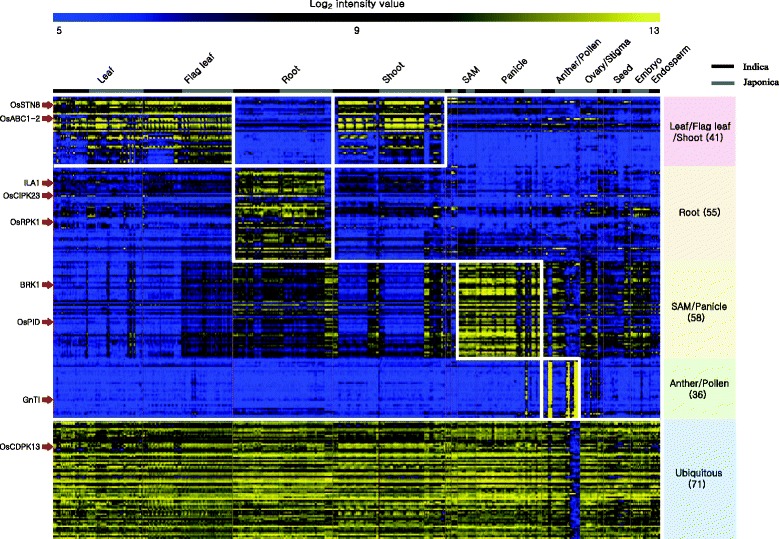
Heatmap analysis of kinase genes showing anatomical tissue-preferred expression pattern. Gene expression pattern reveals five tissue-preferential groups and the total genes in each group is shown in parenthesis. Affymetrix dataset includes the gene expression profiles from both indica and japonica subspecies plant samples. Sample profiling from subspecies are represented using black and grey bars for indica and japonica respectively. Log2-transformed intensity values were taken for generating heatmap and the scale is given above the heatmap. Genes with red arrow pointing the heatmap are the known genes

To assess the biological functions of rice kinases, meta-expression profiles generated from a large collection of microarray-based expression datasets available in NCBI GEO have been used. Global analysis with anatomical data suggested that 55 kinases are predominantly expressed in the roots, 41 in the leaves/shoots, 58 in the callus/panicles, 36 in anthers/pollen and 71 genes are ubiquitously expressed (Fig. 1; Additional file 1: Table S1). Of these, functions for 16 genes have been elucidated through genetics and molecular studies (Table 1). For example, *LOC_Os05g40770* is categorized under root preferred featured anatomical expression group in the current investigation. Previous study revealed that OsRPK1/*LOC_Os05g40770* is expressed in the root tips and encodes a Ca^2 +^-independent leucine-rich-repeat (LRR) receptor-like kinase (RLK). Transgenic rice plants over-expressing *OsRPK1* showed undeveloped adventitious and lateral roots (Zou et al. 2014).

**Fig. 2:**
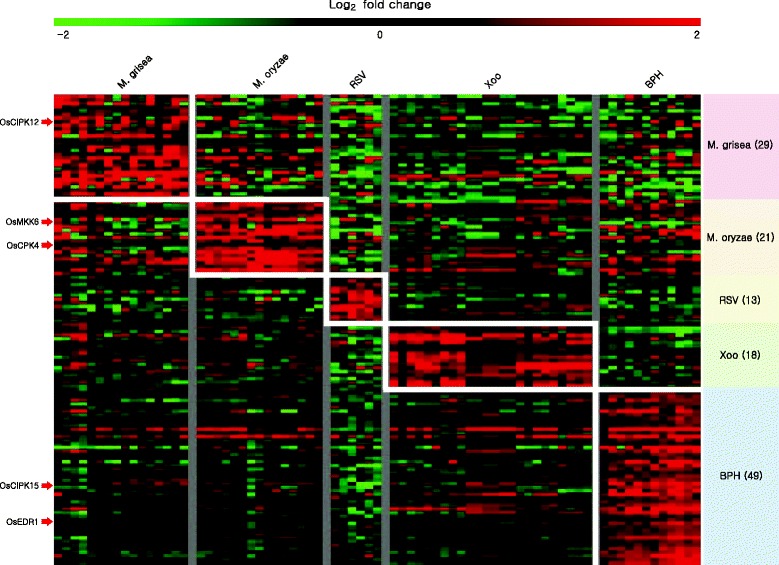
Heatmap analysis of expression patterns for kinase genes related to biotic-stress responses. Biotic stress responsive kinase genes heatmap is generated using log2-transformed fold change values under four stress conditions. Number of stress responsive genes in each condition is shown inside parenthesis after the applied biotic stress. Genes with red arrow pointing the heatmap are the known genes

Biotic stress-related meta-expression data revealed significant upregulation in response to MG (29 kinases), MO (21 kinases), RSV (13 kinases), Xoo (18 kinases), and BPH (49 kinases) (Fig. 2; Additional file 2: Table S2). Of these, functions have been elucidated for eight genes (Table 1). *OsACDR1*/*LOC_Os03g06410*, for which present meta-analysis revealed induction under BPH infection, encodes a putative Raf-like mitogen-activated protein kinase kinase kinase (MAPKKK) and plays a positive regulatory role against fungal infection by modulating defense related gene expression (Kim et al. 2009).

**Fig. 3:**
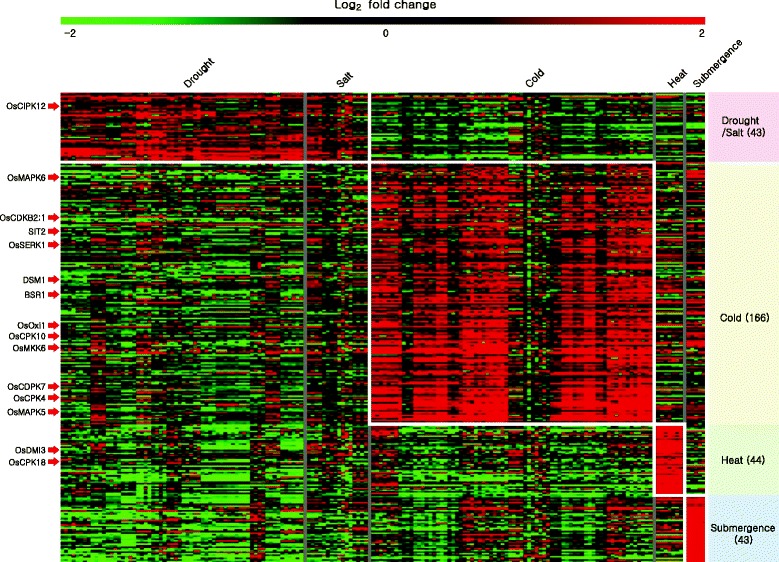
Heatmap analysis of kinase genes related to abiotic-stress responses. Abiotic stress responsive kinase genes heatmap is generated using log2-transformed fold change values under four stress conditions. Number of stress responsive genes in each condition is shown inside parenthesis after the applied abiotic stress. Genes with red arrow pointing the heatmap are the known genes

Abiotic stress-related meta-expression data showed that 43 kinases are induced by drought or salt stress, 166 by low temperature, 44 by heat, and 43 by submergence (Fig. 3; Additional file 3: Table S3). Of these, functions of 23 kinase genes have been elucidated (Table 1). They include calcium-dependent protein kinase *OsCDPK7*/ *LOC_Os04g49510* which acts as positive regulator for stress conditions and confers cold, salt and drought resistance on overexpression (Saijo et al. 2000). Interestingly, we also noted the cold depend induction of *OsCDPK7* in the abiotic stress dataset based analysis and provides a hint about more potential candidates in the featured groups. Furthermore, the genes that are differentially expressed (log2 fold change >2 and *P* < 0.05) in root under heavy metal treatment were analyzed to reveal the potential kinase genes involved in soil toxicity. It was revealed that 21 genes were upregulated whereas expressions of 92 genes were declined under arsenic treatment in soil. Similarly, 44 and 79 genes are induced and declined by cadmium treatment, respectively. More numbers of kinase genes were responsive to chromium: expressions of 142 genes are induced; and that of 206 genes were reduced. In case of lead treatment in soil, 36 genes are upregulated and 54 genes are downregulated (Additional file 4: Table S4).

**Fig. 4:**
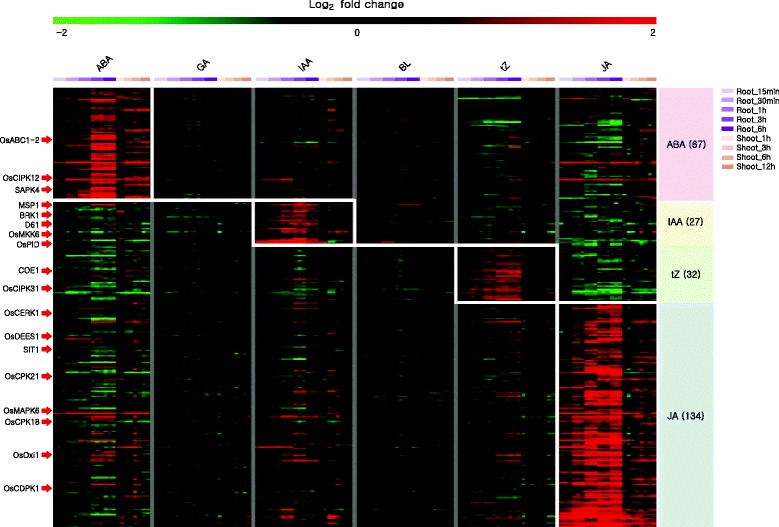
Heatmap analysis of expression patterns for kinase genes in response to hormone treatments. Hormone responsive kinase genes are generated from Agilent log2-transformed fold change hormone dataset, which includes responses from six major hormone treatments at different time intervals in root and shoot. Group of genes induced under specific hormones treatment along with their total number is indicated in parenthesis. Genes with red arrow pointing the heatmap are the known genes

The meta-expression data for hormone responses indicated that 67 kinases are up-regulated upon ABA treatment, 27 by IAA, 32 by tZ, and 134 by JA treatment (Fig. 4; Additional file 5: Table S5). Among them, functions of 18 genes have been characterized previously.

Based on the featured expression data, it is evident that these 947 kinases may serve as potential primary targets for further functional studies, with 261 related to anatomy; 130, to biotic stresses; 296, to abiotic stresses; and 260, to hormone responses.

### Evaluation of functional redundancy or functional dominance for rice kinases with known functions via PCC analysis

**Fig. 5:**
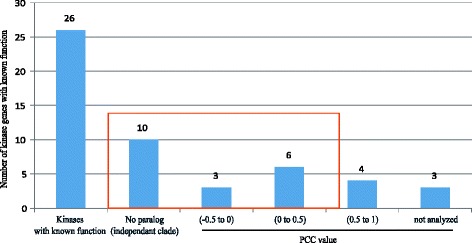
Estimations of functional dominancy for rice kinases, based on meta-expression data. *Y-axis*, number of kinases with known functions; *X-axis*, different groups of kinases with known functions, based on range of PCC values or existence of paralogs in subfamilies

**Fig. 6:**
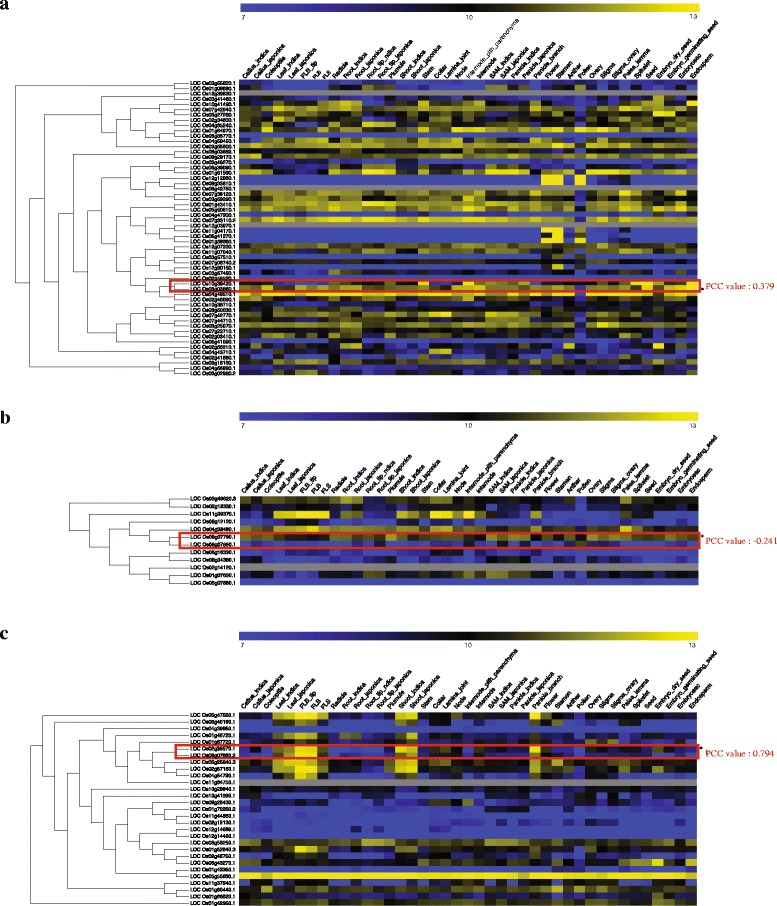
Phylogenomics data for three kinase subfamilies that include members with known functions. Integrating anatomical expression data within context of phylogenic tree suggested functional dominancy of *OsCDPK1* (*LOC_Os03g03660*) with PCC value <0.379 (**a**), *OsBAK1* (*LOC_Os08g07760*) with PC value of −0.241 (**b**), and functional redundancy of *OsABC1-2* (*LOC_Os02g36570*) with PCC value of 0.794 = (**c**). *Red boxes* indicate pairs of kinases used for PCC analysis, gray color indicates the genes without probes in affymetrix array platform,  and asterisks (*) indicate the selected kinases

Regarding morphological or physiological traits, the roles played by 26 kinases have been identified through loss-of-function studies. Pearson correlation coefficient (PCC) can be used to measure the linear dependence between two genes, with a value of ‘1’ representing a perfect, positive correlation. Among paralogous kinase members, PCC values are useful indicators when evaluating functional similarities. PCC values for one to three family members closely linked to each kinase have been generated based on anatomical meta-expression data. These data, summarized in Table 2 and Fig. 5, indicated that 10 kinases exist independently from a clade and another nine have no closely linked members with PCC values >0.5. This explains functional dominancy for the 73 % of kinases with known functions. Therefore, the PCC approach can provide useful estimates of functional redundancy or dominance among family members. In addition, integrating anatomical expression data within the phylogenetics context serves as an effective platform for examining functional dormancy or redundancy within a gene family. Examples of integrated anatomical expression data for subgroups of three kinase families are shown in Fig. 6. Compared with other members, the dominant expression of *OsCDPK1* (*LOC_Os03g03660*) has a PCC value of 0 to 0.5 (Fig. 6a) and predominant expression of *OsBAK1* (*LOC_Os08g07760*) produces a PCC value below zero (Fig. 6B), while *OsABC1-2* (*LOC_Os02g36570*) has redundant expression patterns with PCC values above 0.5 with *LCO_Os09g07660* (Fig. 6c). In the case of *OsABC1-2*, functional studies have used RNAi to repress the expression of *OsABC1-2* to overcome the functional redundancy. The observed phenotype might have been caused by suppression of multiple targets closely linked with *LOC_Os09g07660* in that tree. Further investigation is needed to clarify this possibility.

**Table 2:**
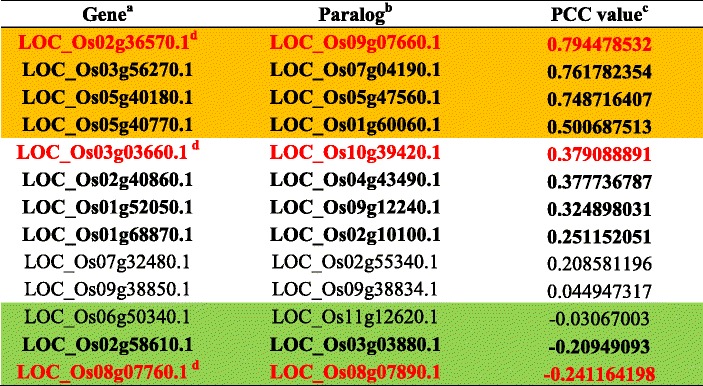
Rice kinase paralogs with known roles based on loss-of-function studies, and PCC values calculated for paralog pairings

^a^Indicates the rice kinase genes which have been functionally characterized and identified from OGRO database (http://qtaro.abr.affrc.go.jp/ogro/table)

^b^Indicates paralogs of rice kinase genes in gene column which are identified from RKD 2.0 databases

^c^Indicates Pierson correlation coefficient (PCC) value between paralog pairs in gene and paralog columns

^d^Indicates rice kinase genes (marked with red letters) used in Figure 6. Brown box has 0.5 > PCC value and is estimated to have redundant roles among paralogs; green one with negative PCC value and uncolored area with 0 < PCC value < 0.5 are estimated to have predominant roles among paralogs

## Conclusions

Of the 1508 kinases featured in RKD 2.0, functions for all except 61 kinases require further examination. This new database will enable researchers to select target kinases more effectively for additional characterization. The predominant expression found among paralogous kinases might be a valuable indicator of functional dominance. Based on loss-of-function analyses and application of PCC values between a kinase and its paralog, we were able to explain the dominancy of 19 kinases with PCC values above 0.5 as well as those that form independent clades in a phylogenetic tree. Primary candidates for loss-of-function studies can be chosen by identifying kinase genes with dominant expression and then comparing them with closely linked family members. These meta-expression data also serve as a source of information about the location and/or conditions that can be applied in subsequent assessments as well as situations in which true functional redundancy may exist. Additional datasets, such as predicted protein–protein interaction networks, further enhance database utility by indicating direct targets regulated by kinases. With the use of gene-indexed mutants that cover at least half of the rice genome, accumulation of diverse omics data will further enhance the functionality of such phylogenomics databases.

## Methods

### Collection of microarray data

Anatomy: Expression data for the anatomical tissues/organs were retrieved from ROAD (Cao et al. 2012).

Abiotic stress: The dataset for abiotic stresses comprised multiple samples from NCBI GEO under the series GSE6901, GSE16108, GSE21651, GSE24048, GSE25176, GSE26280, GSE33204, GSE37940, and GSE38023. Experiment IDs in the Array express dataset (Array Express; https://www.ebi.ac.uk/arrayexpress/. Accessed 2 December 2015) included E-MEXP-2267and E-MEXP-2401. Altogether, 145 individual experiments were parsed for this analysis.

Biotic stress: The GEO series for the biotic stress database involved GSE7256, GSE30941, GSE41798, GSE18361, GSE36272, GSE29967, and GSE11025. In all, 103 individual samples were analyzed under these seven series.

Hormone response: For analyzing hormone-related meta-expression, we relied upon the Agilent 44 K array data GSE39429, as generated by Sato et al. (2013).

### Collection of RNA-Seq data

RNA-Seq anatomical expression dataset consisting of 27 tissues/organs were retrieved from source data developed by Wang et al. (2015). In addition, spatio-temporal transcriptome data of rice root in response to phosphate starvation and recovery were used (Secco et al. 2013).

### PCC analysis

An anatomy dataset was used for estimating PCC values among neighboring genes within a subclade. For each gene, the intensity level of the RGAP gene model probes was fetched and formatted in pairs using in-house scripts. For every pair of genes that showed expression across a range of experiments, a PCC value was calculated using the Microsoft Excel function.

### Heatmap analysis

To examine the expression patterns of kinase genes in various rice tissues/organs as well as in response to abiotic/biotic stresses or hormone treatments, we used a meta-expression analysis based on 1150 Affymetrix array data plus GEO series GSE39429, which utilized the Agilent 44 K microarray platform (GEO platform GPL6854). We then uploaded the log_2_ normalized intensity data (tab-delimited text format) into Multi Experiment Viewer (MEV; http://www.tm4.org/, accessed on October 20, 2015) and created the desired heatmaps (Fig. 1; Additional file 1: Table S1). In addition, we developed a meta-expression database for biotic stress responses to MG, MO, RSV, Xoo, and BPH (Fig. 2; Additional file 2: Table S2), a meta-expression database for responses to drought, salt, cold, heat, and submergence (Fig. 3; Additional file 3: Table S3), and a meta-expression database for responses to six hormones (ABA, GA, IAA, BL, JA, and cytokinin/trans-Zeatin) (Fig. 4; Additional file 5: Table S5). Expression data for selected kinases were presented by heatmaps. For other methods used for construction of RKD 2.0, RKD original version retains the details (Dardick et al. 2007).

## Abbreviations

NCBI GEO, National Center for Biotechnology Information Gene Expression Omnibus; non-TE, non-transposable element; PCC, Pearson correlation coefficient; RGAP, rice genome annotation project database; RKD, rice kinase database; ROAD, rice oligonucleotide array database

## Additional files

Additional file 1: Table S1. Rice kinases with patterns of anatomical expression. (XLSX 25 kb) Additional file 2: Table S2. Rice kinases with abiotic stress responses. (XLSX 19 kb) Additional file 3: Table S3. Rice kinases with biotic stress responses. (XLSX 28 kb) Additional file 4: Table S4 Rice kinases differentially expressed under root heavy metal treatment. (XLSX 62 kb) Additional file 5: Table S5. Rice kinases with hormone responses. (XLSX 25 kb)
